# Alchohol and Longevity

**Published:** 1875-12

**Authors:** 


### ALCOHOL AND LONGEVITY.

Dr. W. T. Harrison, Jr., of Baltimore, submitted an interesting paper to the American Public Health Association last week, on "Alcohol in Relation to Life Insurance." The doctor adopted the cold, practical, unscientific view which men of business take, of the drinking habits of other men, leaving to others the consideration of the effects, moral and physical, upon the individual and upon society, of indulgence in alcoholic drinks. It appears that thirty-four years ago "The United Kingdom Temperance and General Provident Institution" was organized in London, with the intention of restricting its business entirely to teetotalers. The company learned, however, after an extended trial, that the number of this class was not sufficiently large to reduce to the desired limit the cost of insurance, and they were forced either to extend their line or to retire from business. They chose the former alternative, and while still excluding applicants of intemperate habits, they excepted those whose use of alcohol was called temperate. The two classes, however —teetotalers and temperate drinkers —were kept distinct. The statistics of the company down to 1874 give the following results: In the class of teetotalers the actual death rate fell short of the expected rate a fraction over 39 per cent. On the other hand, among the temperate drinkers, the actual death rate, fell short of the expected rate, but to the extent only of one-tenth of one per cent. While believing that the temperate use of alcoholic drinks is not productive of any evil results, and that teetotalism does not furnish the true rule of living, still, says Dr. Harrison, these figures challenge examination, and it behooves us to consider what answer we are to make to an argument based on such facts.

Dr. Harrison believes that a false nomenclature is largely responsible for the blindness of people in general, respecting the abuse in the use of intoxicating liquors. The fact too little insisted on is, that these agents are narcotics as well as stimulants, and nine times out of ten the man who flatters himself that he is stimulated is presenting the symptons of incipient narcosis. Fortunately the instruments of precision with which the physiologist of to-day is armed, enable him to read far clearer than of old, phenomena that are the subjects of his observation. With the thermometer to tell him of a lowered temperature, with a sphygmograph by which the heat may trace the unmistakable evidence of its own growing importance there is no need to wait for so coarse a manifestation as drunkeness to indicate the pernicious influence of alcohol. Long before the cerebro-spinal system shows signs of succumbing, by the uncertainty of the legs and a jabbering of the tongue, the sympathetic nervous system shows unmistakable signs of paralysis,, and the flushed face and quickened pulse, the lowered temperature, the diminished blood pressure, give painful evidence of a profound disorder in the machinery of life.

